# Upregulated IQUB promotes cell proliferation and migration via activating Akt/GSK3β/β‐catenin signaling pathway in breast cancer

**DOI:** 10.1002/cam4.1568

**Published:** 2018-07-02

**Authors:** Kai Li, Yan‐bin Ma, Zun Zhang, Yi‐hao Tian, Xiao‐long Xu, Yan‐qi He, Liu Xu, Yang Gao, Wen‐ting Pan, Wen‐jing Song, Xin He, Lei Wei

**Affiliations:** ^1^ Hubei Provincial Key Laboratory of Developmentally Originated Disease Department of Pathology and Pathophysiology School of Basic Medical Sciences Wuhan University Wuhan Hubei China; ^2^ Department of Anatomy School of Basic Medical Sciences Wuhan University Wuhan Hubei China; ^3^ Hubei Key Laboratory of Tumor Biological Behaviors Department of Breast and Thyroid Surgery Hubei Cancer Clinical Study Center Zhongnan Hospital Wuhan University Wuhan Hubei China

**Keywords:** Akt/GSK3β/β‐catenin signaling pathway, breast cancer, cell migration, cell proliferation, IQ motif and ubiquitin domain containing

## Abstract

Breast cancer was the highest incidence of tumor in women, which seriously threaten women's health. Our previous study found that the expression of IQUB (IQ motif and ubiquitin domain containing) was significantly increased in the development of breast cancer by transcriptome sequencing. However, there were no studies on the mechanism of IQUB in tumorigenesis. Further study showed that IQUB expression was significantly increased in breast cancer, which had a significantly positive correlation with pathological differentiation of breast cancer by tissue microarray analysis. Furthermore, we also discovered that IQUB overexpression could obviously promote the proliferation and migration of MCF‐7 cells and increase the proportion of MCF‐7 cells in S and G2/M phase in vitro study, while knockdown of IQUB caused inhibition of cell proliferation and migration in MDA‐MB‐231 cells and increased the proportion of MDA‐MB‐231 cells in G1 phase. Furthermore, IQUB overexpression or knockdown combined with treatment of Licl or MG‐132 showed that IQUB activated Akt to promote GSK3β phosphorylation, which in turn activated Wnt/β‐catenin signaling pathway in breast cancer cells. Taken together, these results indicated that upregulated IQUB promoted breast cancer cell proliferation and migration via activating Akt/GSK3β/β‐catenin signaling pathway, which played an important part in the tumorigenesis and development of breast cancer.

## 1 INTRODUCTION

According to the Tumor Epidemiology Survey in 2017, breast cancer has become the highest incidence of women's cancer in American, which seriously affects the health of women.^1^ In China, there was an estimated 0.27 million breast cancer cases which leaded to 0.07 million deaths in 2015.^2^ Breast cancer patients mainly died of cancer metastasis; however, the mechanism of breast cancer cells proliferation and metastasis was not clear, which needed further study. Decades of studies had found that there was a close correlation between the development of breast cancer and the activation of oncogenic signaling pathway, such as Wnt/β‐catenin signaling pathway, caused by the inactivation of tumor suppressor gene or the activation of oncogene.^3^ Therefore, looking for new oncogenes and their related signaling pathways wound help understand the mechanism of breast cancer development and provide a scientific basis for the targeted therapy of breast cancer.

In our previous work, we found that IQUB (IQ motif and ubiquitin domain containing) had apparently higher expression in breast cancer than that in normal tissues, suggesting that it may act an important role in the occurrence and development of breast cancer. IQUB was discovered in 2002,^4^ which was located on chromosome 7q31.32 and its encoded protein mainly contained 2 domains, ubiquitin‐like domain and IQ domain. However, there were few studies exploring the role of IQUB in human diseases. Only one tumor‐related study referred to IQUB which found that IQUB was upregulated in gastric cancer tissues by transcriptome sequencing,^5^ but the mechanism was not clear. Combined with our transcriptome sequencing in breast cancer, we speculated that IQUB may act an important role in the development of tumor.

Known as the canonical Wnt signaling pathway, Wnt/β‐catenin signaling pathway regulated the early development of embryo, stem cell self‐renewal, cell division, as well as a variety of biological processes such as tumorigenesis and metastasis.^6^ Aberrant Wnt/β‐catenin signaling pathway frequently occurred in multifarious types of cancers, such as breast cancer^7^ and colorectal cancer.^8^ The overactivation of Wnt/β‐catenin signaling pathway in breast cancer cell leaded to activate the expression of many oncogenic target genes, for instance, c‐myc, and cyclin D1, which induced cell proliferation, migration, and invasion.^9^ Inhibition of Wnt/β‐catenin signaling pathway could inhibit cell proliferation and migration, ultimately inhibiting lung metastasis of breast cancer.^10^ These studies indicated that activation of Wnt/β‐catenin signaling pathway was closely related to the development of breast cancer. β‐catenin was indispensable component of Wnt/β‐catenin signaling pathway whose upregulation leaded to the activation of Wnt/β‐catenin signaling pathway. Axin, APC, GSK3β, and CK1α formed a destruction complex in the cytoplasm.^11^, ^12^ When the destruction complex interacted with β‐catenin, β‐catenin was ubiquitinated and subsequently degraded by cellular proteasome.^13^, ^14^ In addition, as a phosphorylation substrate of Akt, GSK3β could be phosphorylated by Akt at Ser9, which inactivated GSK3β, leading to inhibition of β‐catenin degradation by proteasome.^15^

In this study, we firstly sought to probe the role of IQUB in breast cancer. In addition, we further explored the mechanism of IQUB in promoting breast cancer by activating the Akt/GSK3β/β‐catenin signaling pathway, which would provide a new idea for better comprehension of breast cancer pathogenesis and breast cancer targeted therapy.

## 2 MATERIALS AND METHODS

### 2.1 Human breast cancer samples and tissue microarray

Twenty cases of breast cancer tissues and corresponding paracancerous tissues were collected from Affiliated Zhongnan Hospital of Wuhan University and diagnosed by the Department of Pathology. All patients were informed and agreed. Our research was supported by the Ethics Board of School of Basic Medical Sciences, Wuhan University and was based on all relevant principles of the Declaration of Helsinki. The human breast cancer tissue microarray (the TMA ID: BC081120a) was purchased from Alenabio company (Xi'an, China) which contains 110 cases of breast cancer tissues and corresponding paracancerous tissues.

### 2.2 Plasmids, siRNA, and antibody

The IQUB overexpression plasmid was constructed by cloning the Coding sequence of IQUB gene (NM_001321293.1) into retroviral plasmid pflag‐CMV (Clontech Laboratories Inc.) which was named as pflag‐IQUB, and the negative control as pflag‐NC. The β‐catenin overexpression plasmid was constructed by cloning the Coding sequence of β‐catenin gene (NM_001330729.1), with a 2325‐bp sequence on each flanking side, into retroviral plasmid pEGFP‐C1 (Clontech Laboratories Inc.) which was named as pEGFP‐β‐catenin. The TOP/FOP flash reporter plasmids were purchased from Upstate Biotechnology, containing wild‐type (CCTTTGATC; TOP flash) or mutated (CCTTTGGCC; FOP flash) TCF/LEF DNA binding sites.

The small interfering RNAs of IQUB were provided by GenePharma company in Shanghai, in which siRNA#1 was at nucleotides 282 bp (sense: 5′‐GCCUCAAGAGUCAGAUCAAAC‐3′ and anti‐sense: 5′‐GUUUGAUCUGACUCUUGAGGC‐3′), siRNA#2 at nucleotides 1171 bp (sense:5′‐GCAGAAUACCAUGCUCAAAGA‐3′ and anti‐sense: 5′‐UCUUUGAGCAUGGUAUUCUGC‐3′), and siRNA#3 at nucleotides 2039 bp (sense: 5′‐GCAUAUACCGGUGUCGUAACU‐3′ and anti‐sense: 5′‐AGUUACGACACCGGUAUAUGC‐3′), and the negative siRNA control (sense 5′‐UUCUCCGAACGUGUCACGU‐3′ and anti‐sense 5′‐ACGUGACACGUUCGGAGAA‐3′).

The following primary antibodies were used for Western blotting and immunohistochemistry: IQUB (Biorbyt, UK), flag (Proteintech, USA), GFP (Proteintech), GSK3β (Proteintech), p‐GSK3β(S9) (Cell Signaling Technology, USA), β‐catenin (Proteintech), p‐β‐catenin(S33/S37/T41) (Cell Signaling Technology), c‐myc (Proteintech), cyclin D1 (Proteintech), and β‐actin (Proteintech).

### 2.3 Cell culture and transfection

Human breast cancer cells MCF‐7, MDA‐MB‐231cell lines, and normal breast cells MCF‐10A were purchased from the Cell Bank of Type Culture Collection of the Chinese Academy of Sciences (Shanghai, China). RPMI‐1640 medium or Dulbecco's modified Eagle's medium (DMEM; HyClone, Logan, UT, USA) were used for MDA‐MB‐231 and MCF‐7 cell culture, respectively, supplemented with 10% fetal bovine serum (FBS; Gibco, Milano, Italy), 100 U/mL penicillin and 100 mg/mL streptomycin at 37°C and 5% CO_2_. MCF‐10A cells were cultured in Dulbecco's modified Eagle's medium with F‐12 (DMEM/F‐12; GIBCO) supplemented with 5% horse serum, 20 ng/mL EGF, 0.5 mg/mL hydrocortisone, 100 ng/mL cholera toxin, 10 mg/mL insulin, and 100 U/mL penicillin and 100 mg/mL streptomycin at 37°C and 5% CO_2_ in a humidified incubator.

The transfection of siRNAs and plasmids was performed using Lipofectamine 2000 reagent (Invitrogen Co., Ltd.). The procedures of transfection referred to the manufacturer's instructions. After transfection 48 hours, the cells were used for biological effect detection or detecting the expression level of target genes.

### 2.4 Reverse transcription and quantitative polymerase chain reaction (RT‐qPCR)

Total RNA of cells and tissues was extracted using Trizol reagent (Invitrogen, Carlsbad, CA, USA). Two micrograms RNA was used for first‐strand cDNA synthesis with GeneAmp^™^ RNA PCR Core Kit (Thermo Scientific, USA). Then 2 μL cDNA was used to analyze the expression level of target genes. qPCR (Applied Biosystem Inc.) was used to detect the mRNA expression level. The primers of mRNA for qPCR are IQUB: sense 5′‐TTTCCGCTTTCTGAGCCCTT‐3′ and anti‐sense 5′‐TTAGACATTTTCCTTCACACATACC‐3′, GAPDH: sense 5′‐GGTGAAGGTCGGAGTCAACG‐3′, anti‐sense 5′‐CCATGTAGT TGAGGTCAATGAAG‐3′.

### 2.5 Western Blotting

Proteins of cells were extracted using RIPA lysis buffer, and using a BCA Protein Assay Kit (Beyotime Biotechnology Co., Jiangsu, China) to measure proteins’ concentration. Forty micrograms of total protein was added to each well, running on 12% SDS‐polyacrylamide gel, and then, the proteins were transferred to a PVDF membrane (Millipore, Billerica, MA, USA). The membranes were blocked with Tris‐buffered saline containing 2% BSA for 1 hour and then probed with primary antibodies of target protein, including IQUB (dilution of 1:1000), flag (dilution of 1:2000), GFP (dilution of 1:2000), β‐catenin (dilution of 1:1000), p‐β‐catenin(dilution of 1:500), GSK3β(dilution of 1:1000), p‐GSK3β(dilution of 1:500), cyclin D1(dilution of 1:1000), c‐myc(dilution of 1:1000) and β‐actin(dilution of 1:5000) at 4°C overnight, then incubated with the corresponding secondary antibody (1:5000, Proteintech) for 1 hour at room temperature, and finally detected by ECL reagents (Tanon, Shanghai, China). The optical density of bands was measured by a computer‐assisted imaging analysis system (Tanon), and the relative protein expression levels were normalized to β‐actin.

### 2.6 Cell proliferation assay

CCK‐8 and colon formation assays were used to detect the proliferative capacity of breast cancer cells. For CCK‐8 assay, cells were seeded in 96‐well plates at a density of 1 × 10^3^ per well, and CCK‐8 solution was added at 0, 12, 24, and 48 hours, respectively, and the value of OD450 was measured after 2 hours. For colon formation assay, 200 cells were seeded in 6‐well plates. After 1 week, the cells were fixed and stained and then counted the colon number using a microscope.

### 2.7 Cell migration assay

The ability of cell migration was detected by wound healing and transwell assays. For the wound healing assay, cells were cultured in 6‐well plates for 24 and 48 hours, respectively. A pipette tip (200 μL) was used to make a straight scratch. At 0, 24 and 48 hours, cell wound images were taken by a microscope. For the transwell assay, 2 × 10^4^ cells ware placed on the upper surface of the chamber (Corning, USA) and 500 μL medium containing 10% FBS was added to the lower chamber. After 24 hours, the cells ware fixed and stained then counted the cell number in lower surface of the chamber using a microscope. Three random fields are counted for each experiment.

### 2.8 Cell cycle analysis

Cell cycle was quantified by flow cytometry. Briefly, cells were trypsinized and collected and then fixed with pre‐cooled anhydrous ethanol for 30 minutes. Removing ethanol, per tube was added with 200 μL PBS and 2 μL RNase (0.25 mg/mL) (incubate at 37°C for 30 minutes); then, the cell pellet was mixed with 0.5 mL of 50 μg/mL PI solution, staining for 30 minutes at room temperature in dark. The fluorescence intensity was analyzed by flow cytometry.

### 2.9 TOP/FOP flash analysis

Breast cancer cells were cultured in 24‐well plates. When reaching logarithmic growth phase, cells were transfected with pflag‐IQUB overexpression plasmid or negative control plasmid (400 ng/well), siR‐IQUB, or negative control siR‐NC (15 pmol/well), along with TOP Flash or FOP Flash plasmid (400 ng/well) and pRL‐TK plasmid (10 ng/well) using Lipofectamine 2000 reagent according to the manufacturer's instruction. After 48 hours, firefly luciferase activity and renilla luciferase activity were measured by a dual luciferase reporter assay kit (Promega) according to the manufacturer's instructions. Each experiment was performed independently and repeated 3 times.

### 2.10 Immunohistochemistry

Slides ware deparaffinized and dehydrated by graded alcohol. Antigen retrieval was performed by boiling the slides in 1 mmol/L sodium citrate buffer pH 6.0 and then at sub‐boiling temperature for 20 minutes. Endogenous peroxidase activity was blocked by incubating the slides with 3% H_2_O_2_ for 10 minutes. Slides were washed in PBS, blocked with serum for 30 minutes, and incubated with a rabbit antibody for IQUB (1:400 dilution; Biorbyt) at 4°C overnight in a humidified container. After washing 3 times with PBS, the corresponding anti‐rabbit secondary antibody was incubated with a 1:300 dilution for 2 hours at 37°C. Staining was performed using 3, 3‐diaminobenzidine (DAB; Vector laboratories) for 2 minutes, and the colored reaction was terminated using distilled water. Slides ware counterstained with hematoxylin and Eosin Staining Kit (Beyotime Biotechnology Co.) to visualize cell nuclei, followed by bluing in running tap water. Finally, the slides were dehydrated, dipped in xylene and mounted. The Pannoramic MIDI automatic digital slide scanner (3DHISTECH Ltd., Budapest, Hungary) was used for image processing and quantifications. The protein expression levels of IQUB were measured by the intensity of immunohistochemical staining which could be quantified using an IHC analyzer in Image J software.^16^

### 2.11 Statistical analysis

The data were expressed as the mean ± standard deviation (SD). Differences between groups were analyzed using one‐way ANOVA analysis. χ^2^‐tests were performed to determine significance of the relationship between expression of IQUB and clinicopathologic features in breast cancer tissue microarrays. *P* *<* .05 was considered statistically significant.

## 3 RESULTS

### 3.1 IQUB mRNA and protein levels were increased in breast cancer, which was positively correlated with pathological differentiation

**Figure 1:**
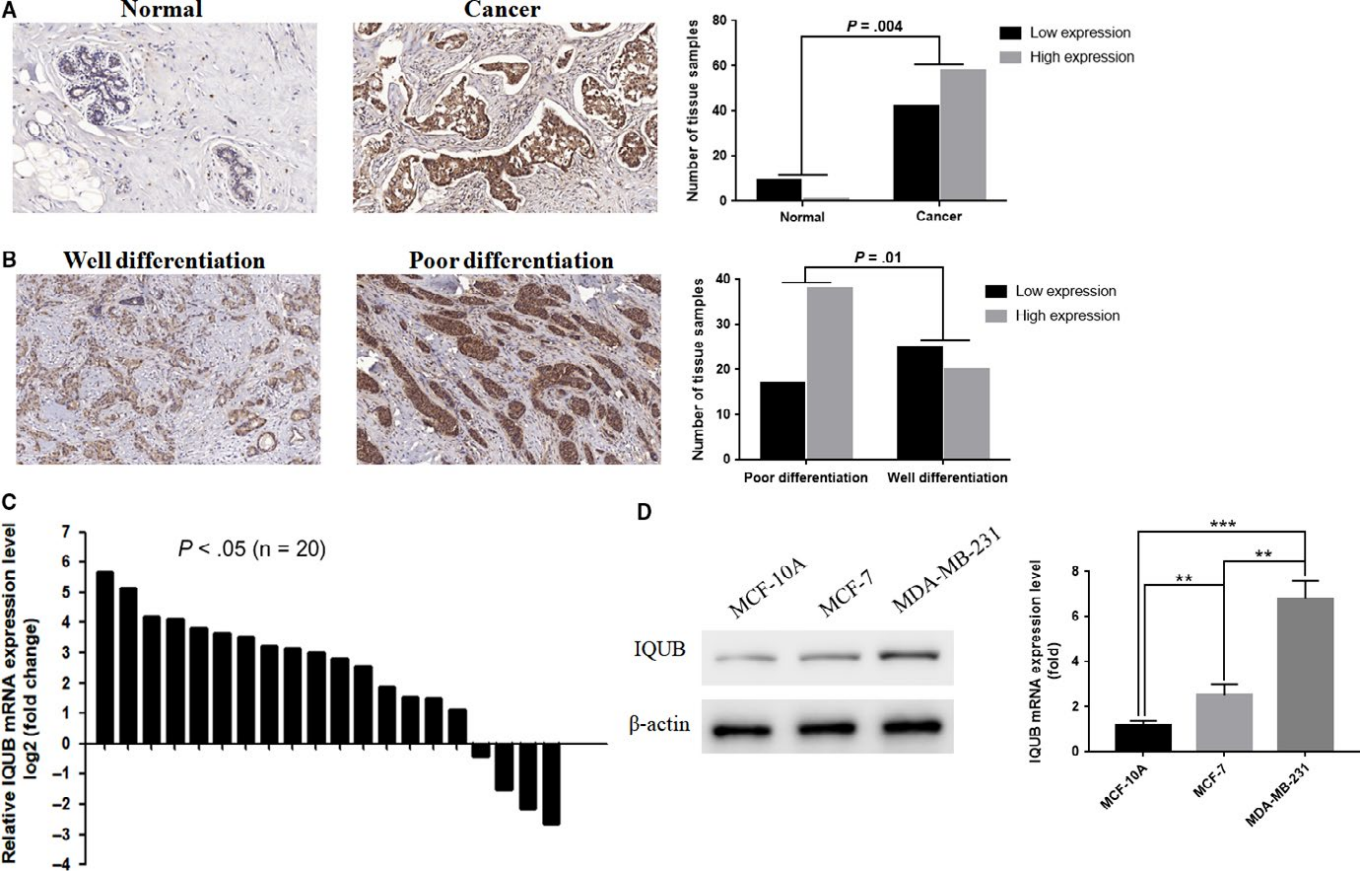
IQUB is significantly upregulated in human breast cancer tissues and cells. A, IQUB protein expression (the brown staining areas) was increased in breast cancer, which was detected in 110 cases of human breast cancer tissue microarray by immunohistochemistry. B, The expression of IQUB in poor differentiation of breast cancer tissues was higher than that in well differentiation of breast cancer tissues. C, IQUB mRNA expression was upregulated in breast cancer tissues (16/20) than paired normal breast tissues which was analyzed by RT‐qPCR (*x*‐axis represent 20 pairs of tissue samples). D, IQUB mRNA and protein expression were detected by RT‐qPCR and Western blotting in normal breast cell line MCF‐10A and breast cancer cell lines MCF‐7 and MDA‐MB‐231. Values represent the mean ± SD from 3 independent measurements. **P <* *.01, ***P <* *.001

To investigate the expression of IQUB protein between normal breast tissues and breast cancer tissues, we examined breast cancer tissue microarray, which included 100 cases of breast cancer tissues by immunohistochemistry. As was shown in Table 1 and Figure 1A,B, compared to normal breast tissue, the expression of IQUB protein (the brown staining areas) was increased significantly in breast cancer tissues (*P *=* *.004), which was higher in poorly differentiated cancer than well‐differentiated cancer (*P *=* *.01). Then, we detected IQUB mRNA expression in 20 pairs of breast cancer tissues and adjacent normal tissues by RT‐qPCR. The result indicated that compared to adjacent tissues, the expression of IQUB mRNA was significantly increased in 16 cases of breast cancer tissues (*P *<* *.05) (Figure 1C). In addition, the mRNA and protein expression of IQUB in breast cancer cells MDA‐MB‐231 and MCF‐7 were significantly higher than that of normal breast cells MCF‐10A, while the expression of IQUB in the more aggressive MDA‐MB‐231 cells was even higher than that in the less aggressive MCF‐7 cells (*P *<* *.01) (Figure 1D).

**Table 1:** IQUB expression and clinicopathologic characteristics of breast cancer TMA

	Cases (n = 100)	IQUB protein expression in cancer tissue (n)		*P* value
		Low expression	High expression	
Age				
<60	86	36	50	>.05
≥60	14	6	8	
T stage				
T1	11	6	5	>.05
T2	66	30	36	
T3–4	23	6	17	
N stage				
N0	78	33	45	>.05
N1–2	22	9	13	
TNM staging				
I	10	6	4	>.05
II	72	29	43	
III–IV	18	7	11	
Differentiation				
Well/moderate	45	25	20	.01
Poor	55	17	38	

*P* values were based on χ^2^‐test, *P* < .05 was considered statistically significant.

### 3.2 IQUB could promote proliferation of breast cancer cells via accelerating G1/S transition

**Figure 2:**
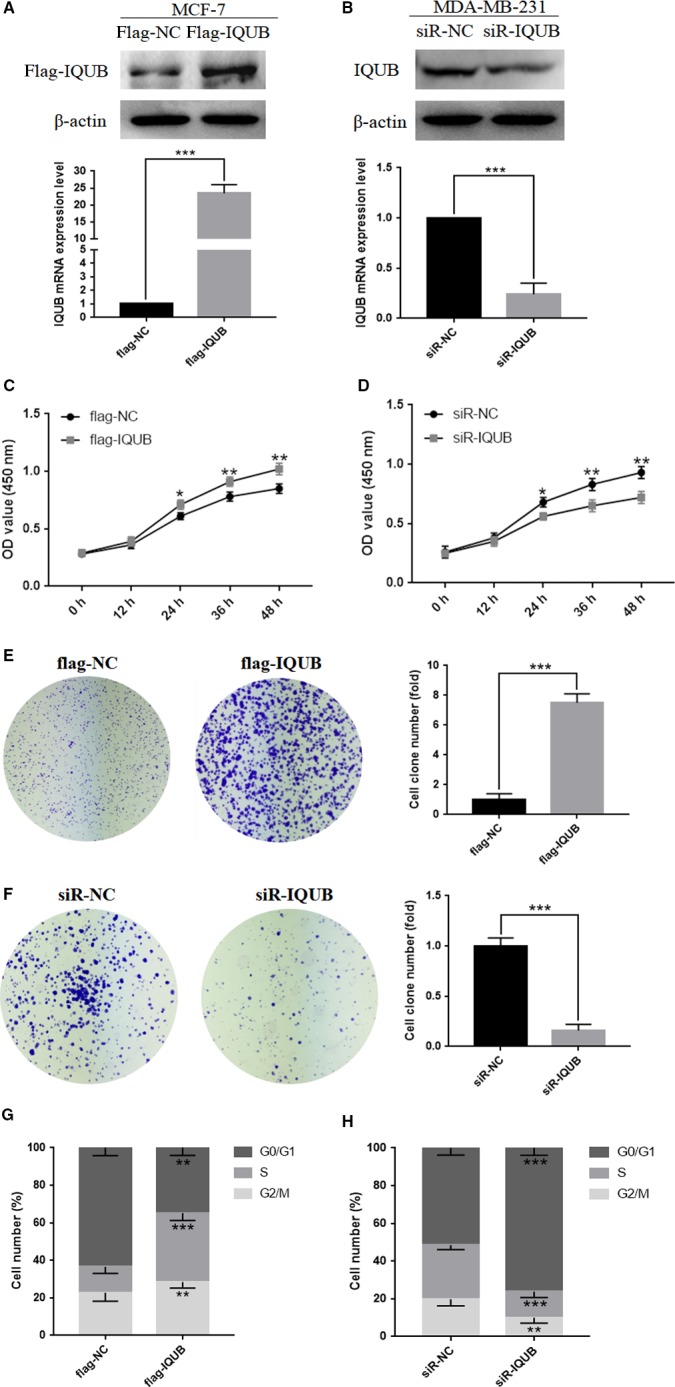
IQUB promotes proliferation of breast cancer cells, and accelerating G1/S transition. A, MCF‐7 cells were transfected with flag‐IQUB overexpression plasmid or flag‐NC negative control vector and detected by RT‐qPCR and Western blotting. B, MDA‐MB‐231 cells were transfected with small interfering RNA of IQUB (siR‐IQUB) or negative control (siR‐NC) and detected by RT‐qPCR and Western blotting. C, IQUB overexpression promotes proliferation of MCF‐7 cells which was detected by the CCK‐8 assay. D, IQUB knockdown inhibits proliferation of MDA‐MB‐231 cells which was detected by the CCK‐8 assay. E, IQUB overexpression enhances proliferation of MCF‐7 cells which was detected by the colon formation assay. F, IQUB knockdown inhibits proliferation of MDA‐MB‐231 cells which was detected by the colon formation assay. G, IQUB overexpression increases the portion of MCF‐7 cells in S and G2/M phase which was detected by flow cytometry. H, IQUB knockdown increases the portion of MDA‐MB‐231 cells in G1 phase, and decreases the portion of cells in S and G2/M phase which was detected by flow cytometry. Values represent the mean ± SD from 3 independent measurements. *P <* *.05, **P <* *.01, ***P <* *.001

In vitro study, the biological functions of IQUB in breast cancer cells were detected by gain‐ and loss‐of‐expression strategy. We firstly constructed IQUB overexpression plasmids (pflag‐IQUB) and small interfering RNA (siR‐IQUB) and then transfected them into MCF‐7 and MDA‐MB‐231 cells. Then, the proliferation of breast cancer cell was detected by CCK‐8 assay and clone formation assay (Figure 2A,B). The results indicated that overexpression of IQUB could significantly promote proliferation of MCF‐7 cells, while knocking down IQUB showed the opposite effect in MDA‐MB‐231 cells (Figure 2C‐F). In addition, through analysis of flow cytometry, we found that overexpression of IQUB significantly increased the proportion of MCF‐7 cells in S/G2 phase, while knockdown of IQUB arrested MDA‐MB‐231 cells in G1 phase (Figure 2G,H). These results showed that IQUB could promote breast cancer cells proliferation via accelerating G1/S transition.

### 3.3 IQUB could promote migration of breast cancer cells

**Figure 3:**
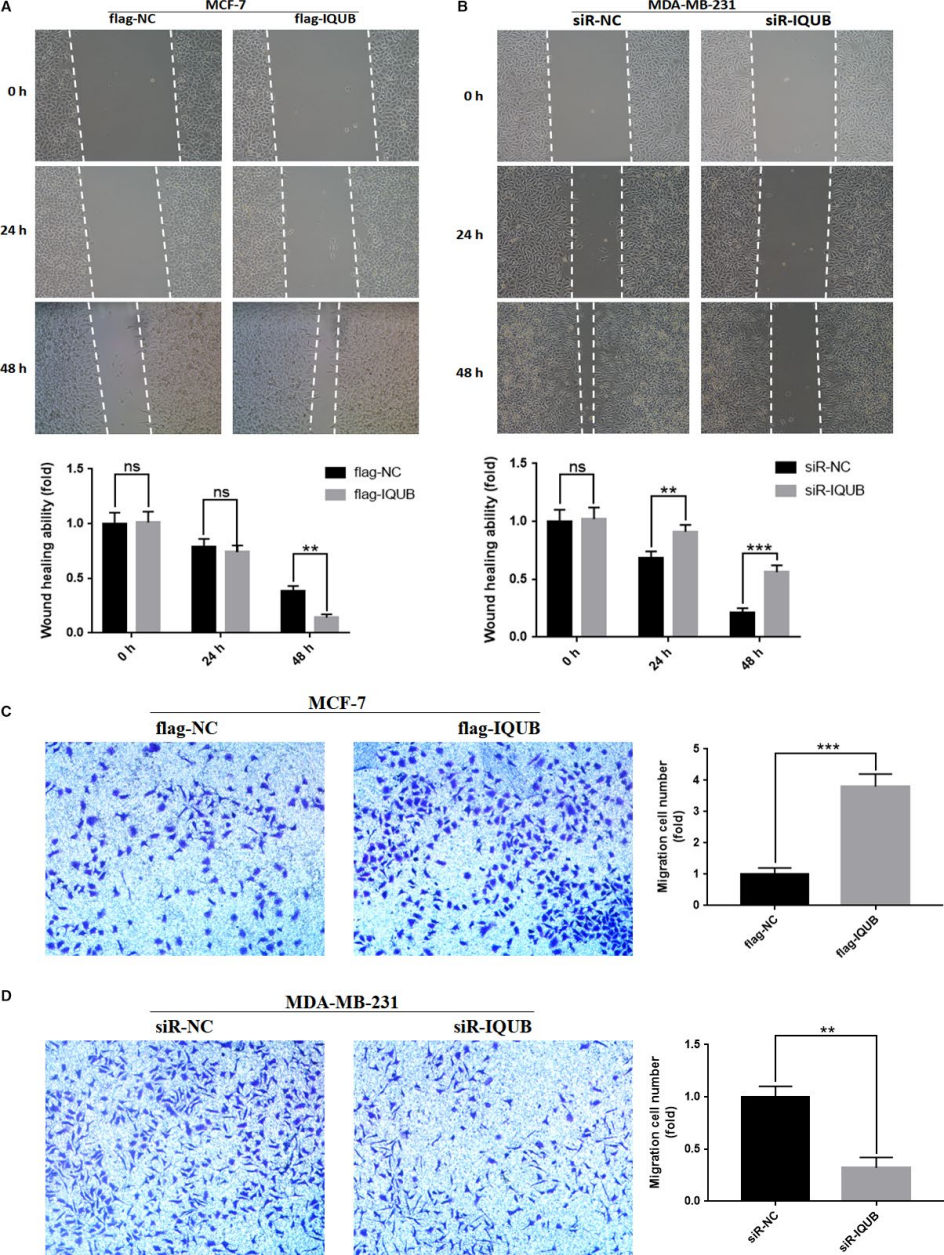
IQUB promotes migration of breast cancer cells. A, IQUB overexpression promotes migration of MCF‐7 cells which was detected by Wound healing assay. B, IQUB knockdown inhibits migration of MDA‐MB‐231 which was detected by Wound healing assay. C, IQUB overexpression enhances migration of MCF‐7 cells which was detected by transwell assay. D, IQUB knockdown suppresses migration of MDA‐MB‐231 which was detected by transwell assay. Values represent the mean ± SD from 3 independent measurements. **P <* *.01, ***P <* *.001, ns represents no statistical significance

To test the effect of IQUB on cell migration, we transfected IQUB overexpression plasmid flag‐IQUB and negative control flag‐NC, small interfering RNA of IQUB siR‐IQUB and negative control siR‐NC into breast cancer cells. Then Wound healing assay and transwell assay were carried out to detect the migration of breast cancer cells. The results of Wound healing assay revealed that compared with the flag‐NC transfected group, the migration ability of MCF‐7 cells was significantly enhanced at 48 hours after transfection with flag‐IQUB, while there was no significant difference at 24 hours after transfection (Figure 3A). Compared with control (siR‐NC), knockdown of IQUB (siR‐IQUB) significantly weakened the migration ability of MDA‐MB‐231 cell at 24 and 48 hours after transfection (Figure 3B). In addition, the results of transwell assay revealed that overexpression of IQUB (flag‐IQUB) significantly promoted migration of MCF‐7 cells, whereas knockdown of IQUB (siR‐IQUB) significantly inhibited migration of MDA‐MB‐231 cells (Figure 3C,D). According to these results, overexpression of IQUB could significantly promote migration of breast cancer cells.

### 3.4 IQUB activated Wnt/β‐catenin signaling pathway in breast cancer cells

**Figure 4:**
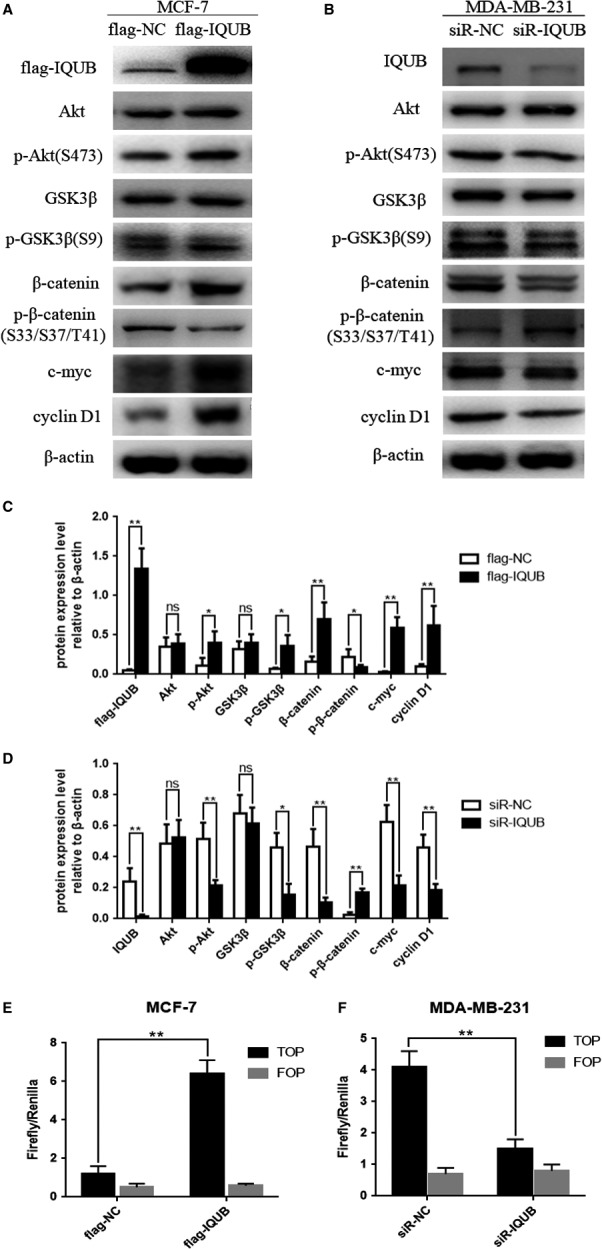
IQUB activates Wnt/β‐catenin signaling pathway in breast cancer cells. A, The effect of transfecting with flag‐IQUB overexpression plasmid or flag‐NC negative control vector on the protein levels of flag‐IQUB, Akt, p‐Akt(S473), β‐catenin, p‐β‐catenin (S33/S37/T41), GSK3β, p‐GSK3β(S9), c‐myc, cyclin D1 in MCF‐7 cells. B, The effect of transfecting with small interfering RNA of IQUB (siR‐IQUB) or negative control (siR‐NC) vector on the protein levels of IQUB, Akt, p‐Akt(S473), β‐catenin, p‐β‐catenin (S33/S37/T41), GSK3β, p‐GSK3β(S9), c‐myc, cyclin D1 in MDA‐MB‐231 cells. C, The protein levels of flag‐IQUB, Akt, p‐Akt, β‐catenin, p‐β‐catenin, GSK3β, p‐GSK3β, c‐myc, cyclin D1 relative to β‐actin in MCF‐7 cells based on statistical analysis. D, The protein levels of IQUB, Akt, p‐Akt, β‐catenin, p‐β‐catenin, GSK3β, p‐GSK3β, c‐myc, cyclin D1 relative to β‐actin in MDA‐MB‐231 cells based on statistical analysis. E, IQUB overexpression increases the activity of Wnt/β‐catenin signaling pathway which was detected by TOP/FOP flash assay in MCF‐7 cells. F, IQUB knockdown inhibits the activity of Wnt/β‐catenin signaling pathway which was detected by TOP/FOP flash assay in MDA‐MB‐231 cells. Values represent the mean ± SD from 3 independent measurements. *P <* *.05, **P <* *.01, ns represents no statistical significance

As one of the most classical signaling pathways in tumorigenesis, the role of Wnt/β‐catenin signaling pathway in the tumorigenesis had been repeatedly studied. The overactivity of Wnt/β‐catenin signaling pathway was often found in tumorigenesis and progression of breast cancer, which could significantly enhance the proliferation and migration ability of tumor cells. Therefore, we examined the association between IQUB and Wnt/β‐catenin signaling pathway in breast cancer cells. As was shown, overexpression of IQUB in MCF‐7 cells could significantly promote the expression of β‐catenin, p‐GSK3β (S9), and downstream target genes cyclin D1 and c‐myc while inhibited the expression of p‐β‐catenin (S33/S37/T41), but did not affect protein expression of GSK3β (Figure 4A,C). Consistent with the previous results, knockdown of IQUB in MDA‐MB‐231 cells notably inhibited the expression of β‐catenin, p‐GSK3β (S9), cyclin D1, and c‐myc, while increased the expression of p‐β‐catenin (S33/S37/T41), but also did not affect the expression of GSK3β (Figure 4B,D). In addition, TOP/FOP flash assay, which was used to determine the activity of Wnt/β‐catenin signaling pathway, was carried out to detect the association between IQUB and the activation of Wnt/β‐catenin signaling pathway. In MCF‐7 cells, overexpression of IQUB significantly increased TOP flash reporter activity (Figure 4E), while knockdown of IQUB in MDA‐MB‐231 cells significantly inhibited TOP flash reporter activity (Figure 4F). These results indicated that IQUB overexpression in breast cancer cells could activate Wnt/β‐catenin signaling pathway.

### 3.5 IQUB promoted proliferation and migration of breast cancer cell via activating Akt/GSK3β/β‐catenin signaling pathway

**Figure 5:**
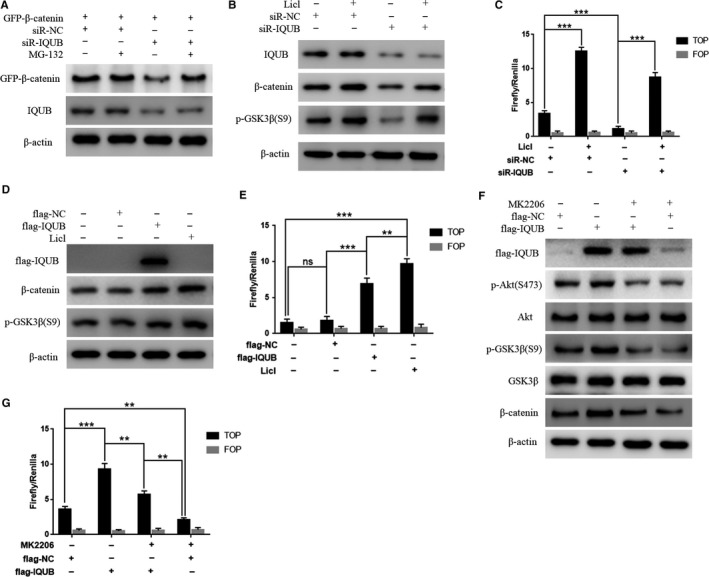
IQUB activates Wnt/β‐catenin signaling pathway via Akt/GSK3β pathway. A, Proteasomes inhibitor MG‐132 reverses the effect of IQUB knockdown on β‐catenin degradation. B, Inhibiting GSK3β kinase activity by Licl reduces inhibition of β‐catenin expression by IQUB knockdown. siR‐IQUB or siR‐NC was transfected into MDA‐MB‐231 cells. 36 h after transfection, the cells were treated with 30 mmol/L Licl for 12 h and then harvested for Western blotting analysis to detect the expression of GFP‐β‐catenin, p‐GSK3β (S9) and IQUB. C, Licl reverses the effect of IQUB knockdown on inhibitory activity of Wnt/β‐catenin signaling pathway. siR‐IQUB or siR‐NC was transfected into MDA‐MB‐231 cells. 36 h after transfection, the cells were treated with 30 mmol/L Licl for 12 h and harvested for luciferase activity assay. D, MDA‐MB‐231 cells were transfected with flag‐NC and flag‐IQUB, treated with Licl for 48 h and harvested for Western blot assay. E, MDA‐MB‐231 cells were transfected with flag‐NC and flag‐IQUB, treated with Licl for 48 h and harvested for TOP/FOP flash assay. F, Akt inhibitor MK2206 (0.1 μmol/L) reduces effect of IQUB overexpression on the expression of p‐Akt (S473), p‐GSK3β (S9), and β‐catenin. G, Akt inhibitor MK2206 (0.1 μmol/L) reduces effect of IQUB overexpression on the activity of Wnt/β‐catenin signaling pathway. **P <* *.01, ***P <* *.001, ns represents no statistical significance

**Figure 6:**
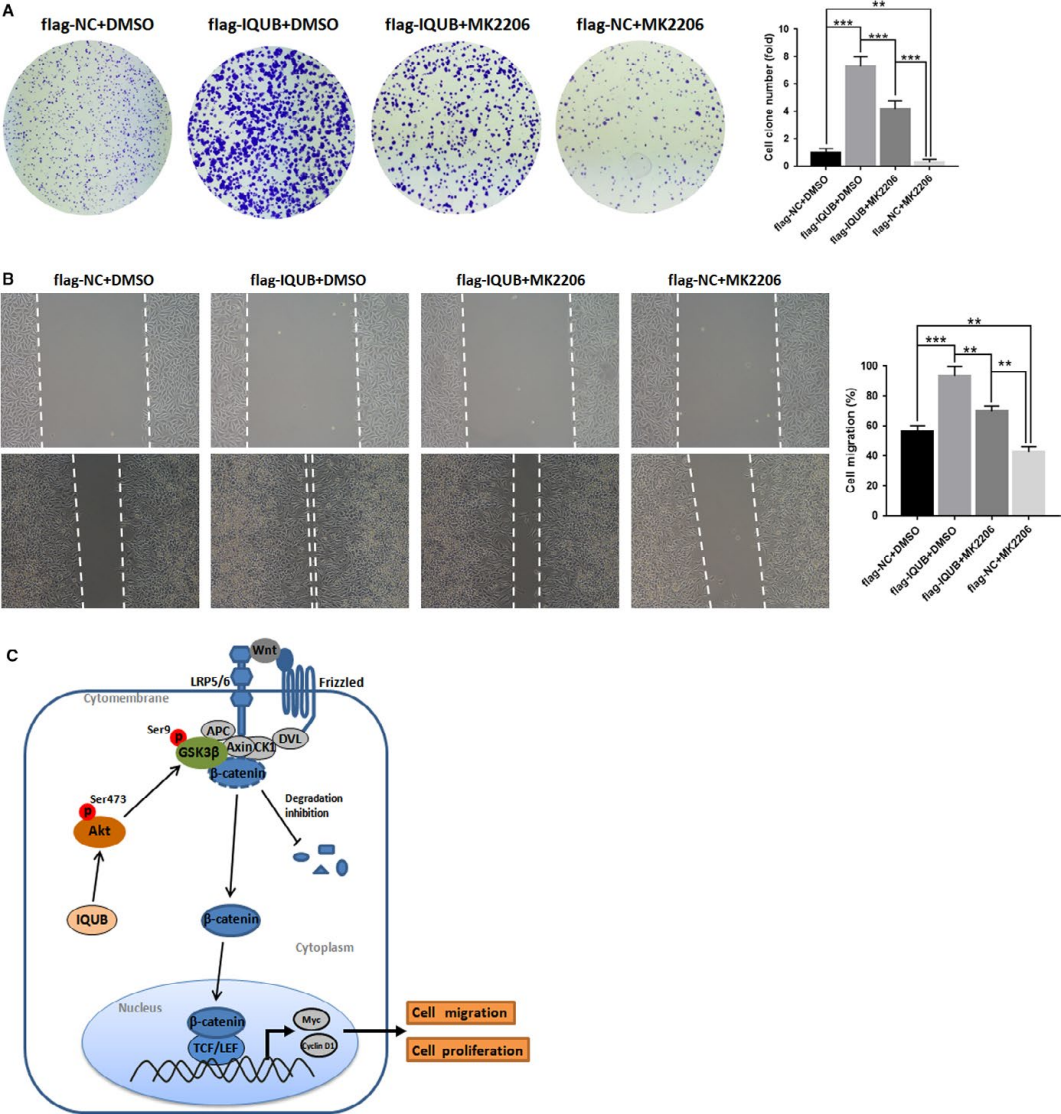
IQUB promotes proliferation and migration of breast cancer cells via Akt/GSK3β/β‐catenin signaling pathway. A, Akt inhibitor MK2206 (0.1 μmol/L) reduces effect of IQUB overexpression on promoting proliferation of MDA‐MB‐231 cells. B, Akt inhibitor MK2206 (0.1 μmol/L) reduces effect of IQUB overexpression on promoting migration of MDA‐MB‐231 cells. C, Working model for the regulation of proliferation and migration of breast cancer cells by IQUB via Akt/GSK3β/β‐catenin signaling pathway. The upregulated IQUB promotes proliferation and migration of breast cancer cells via activating Akt/GSK3β/β‐catenin signaling pathway. **P < .01, ***P < .001

In the present study, we also noticed that overexpression of IQUB significantly upregulated the expression of p‐Akt (S473), while knockdown of IQUB showed the opposite effect (Figure 4A,B). It was known that GSK3β was a phosphorylation substrate of Akt. When Akt was activated by phosphorylation at Ser473, it could promote phosphorylation of GSK3β at Ser9, which leaded to inactivation of GSK3β.^17^ GSK3β was the key factor to promote the phosphorylation of β‐catenin at Ser33 and Ser37, which leaded β‐catenin to be degraded by proteasome.^6^ The inactivation of GSK3β by Ser9 phosphorylation promoted accumulation of β‐catenin and ultimately activated the Wnt/β‐catenin signaling pathway.^18^ Firstly, we treated MDA‐MB‐231 cells with MG‐132, which could inhibit the activity of protease, and then observed the effect of IQUB knockdown on β‐catenin expression and the activity of Wnt/β‐catenin signaling pathway. The results showed that MG‐132 could significantly reverse the inhibition of β‐catenin expression and Wnt/β‐catenin signaling pathway by IQUB knockdown (Figure 5A), suggesting that IQUB could inhibit β‐catenin degradation, and thereby activated Wnt/β‐catenin signaling pathway. In addition, we treated MDA‐MB‐231 cells with Licl, an inhibitor of GSK3β, and simultaneously knocked down IQUB. We found that Licl significantly reversed the inhibition of β‐catenin expression and Wnt/β‐catenin signaling by IQUB knockdown (Figure 5B,C), suggesting that IQUB regulated Wnt/β‐catenin signaling pathway by GSK3β pathway. Furthermore, we found that overexpression of IQUB shared similar effect with Licl, upregulating the expression of β‐catenin and p‐GSK3β (S9), and activating Wnt/β‐catenin signaling pathway (Figure 5D,E). To investigate the role of Akt in IQUB regulation of Wnt/β‐catenin signaling pathway, we treated MDA‐MB‐231 cells with Akt inhibitor MK2206 and observed the effect of IQUB overexpression on p‐GSK3β and β‐catenin expression and activity of Wnt/β‐catenin signaling pathway. The results indicated that MK2206 could significantly inhibit the upregulation of p‐GSK3β (S9) and β‐catenin, as well as the activation of Wnt/β‐catenin signaling pathway by IQUB overexpression (Figure 5F,G). In addition, we also found that MK2206 could significantly inhibit the effect of IQUB overexpression on promoting proliferation and migration of breast cancer cells by clone formation assay and Wound healing assay (Figure 6A,B). These results showed that upregulated IQUB could promote proliferation and migration of breast cancer cells via activating the Akt/GSK3β/β‐catenin signaling pathway.

## 4 DISCUSSION

There was no study on the mechanism of IQUB in tumorigenesis. Only one study mentioned that IQUB expression was increased in gastric cancer by transcriptome sequencing.^5^ In our study, we noticed that the expression of IQUB in breast cancer tissues was not only significantly increased, but also positively correlated with the pathological differentiation of breast cancer, suggesting that IQUB may have a bearing on the malignant progression and prognosis of breast cancer. In vitro study, overexpression of IQUB could significantly enhance the proliferation and migration ability of breast cancer cells, whereas knockdown of IQUB showed the opposite effect. These results suggested that IQUB acted as oncogene in the development of breast cancer.

Uncontrolled proliferation of cells was one of the most basic features of cancer, which was also required to cancer invasion and metastasis.^19^ Cell cycle reflected the process of cell division and proliferation, including G0, G1, S, G2, and M phases.^20^ G1 phase was the preparation period, once the transition from G1 phase to S phase finished, the cell cycle would not stop until the cell division was completed.^21^ Therefore, an increase in the proportion of cells at S and G2/M phase represented an enhanced proliferation of cells.^22^ Cyclin‐dependent kinases (CDKs), such as CDK4 and CDK6, were a family of protein kinases that were first discovered for their role in regulating the cell cycle.^23^, ^24^ Cyclin D1 forms protein complex with CDK4 or CDK6, the activity of which is necessary for cell cycle G1/S transition.^25^ The upregulation of cyclin D1 expression could accelerate the cell cycle progression and eventually lead to tumor cell proliferation.^26^, ^27^ According to the present study, we found that IQUB could positively regulate the expression of cyclin D1 in breast cancer cells. Furthermore, it was found by flow cytometry that IQUB overexpression induced G1/S transition in MCF‐7 cells, while IQUB knockdown decreased proportion of MDA‐MB‐231 cells in S/G2 phase, suggesting that IQUB could promote proliferation of breast cancer cells by accelerating G1/S transition. Besides that, we also found that IQUB significantly upregulated expression of c‐myc. Interestingly, cyclin D1 and c‐myc were both target genes of Wnt/β‐catenin signaling pathway.^28^ Therefore, we hypothesized that IQUB activated Wnt/β‐catenin signaling pathway and thus played a role in promoting the proliferation and migration of breast cancer cells. In addition, we found that overexpression of IQUB significantly upregulated the expression of β‐catenin, while knockdown of IQUB inhibited the expression of β‐catenin. In addition, the overexpression of IQUB significantly increased the activity of Wnt/β‐catenin signaling pathway, while IQUB knockdown significantly reduced the activity of Wnt/β‐catenin signaling pathway by TOP/FOP flash assay. In conclusion, our study indicated that IQUB promoted the proliferation and migration of breast cancer cells via activating Wnt/β‐catenin signaling pathway. However, there were no studies explored the mechanism of IQUB regulating Wnt/β‐catenin signaling pathway.

For the Wnt/β‐catenin signaling pathway, Wnt protein interacted with the Frizzled family receptor on the cell membrane, and then disheveled (DVL) protein in the cytoplasm received biological signals and continued to transmit, resulting in the accumulation of β‐catenin in the cytoplasm, eventually leading β‐catenin to enter the nucleus to interact with TCF/LEF family of proteins to form a transcriptional activation complex, finally activated a series of cell proliferation and migration‐related target genes.^29^ Axin, APC, GSK3β, and CK1α in the cytoplasm formed degradation complexes when Wnt signaling pathway was inactivated.^13^ When the degradation complex interacted with β‐catenin, β‐catenin was phosphorylated and ubiquitinated, followed by degradation by intracellular proteasomes.^14^ The effect of degradation complex mainly depended on the kinase activity of GSK3β. Phosphorylation of β‐catenin at Ser33 and Ser37 by GSK3β could finally leading to β‐catenin degradation, and thereby inhibited Wnt/β‐catenin signaling pathway.^30^ Therefore, reduced kinase activity of GSK3β will activate Wnt/β‐catenin signaling pathway.^18^ It was known that phosphorylation of GSK3β at Ser9 would lead to inactivation of GSK3β. In addition, GSK3β was a phosphorylation substrate of Akt.^17^ Activation of Akt promoted the phosphorylation of GSK3β at Ser9, which in turn inhibited the degradation of β‐catenin and activated Wnt/β‐catenin signaling pathway.^31^ In this study, we found that IQUB overexpression increased the expression of p‐Akt, p‐GSK3β, and inhibited p‐β‐catenin, whereas IQUB knockdown showed the opposite effect. Take all these results into consideration, IQUB may activate Wnt/β‐catenin signaling by activating Akt/GSK3β pathway.

Furthermore, we found that Licl, a GSK3β inhibitor, could significantly reverse the inhibitory effect of IQUB knockdown on the expression of β‐catenin and activity of Wnt/β‐catenin signaling pathway. Furthermore, IQUB overexpression showed a similar effect with Licl, which both acted as GSK3β inhibitor to activate Wnt/β‐catenin signaling pathway. Moreover, Akt inhibitor MK2206 could significantly inhibit the effect of IQUB overexpression on upregulating p‐GSK3β and β‐catenin and activating Wnt/β‐catenin signaling pathway. These results indicated that IQUB could activate Wnt/β‐catenin signaling pathway through Akt/GSK3β pathway. Furthermore, it was interesting to note that inhibition of Akt activity could not completely abolish the effect of IQUB overexpression on Wnt/β‐catenin signaling pathway activity, suggesting that IQUB may be able to regulate Wnt/β‐catenin signaling pathway via another mechanism. Finally, we found that MK2206 also reversed the effect of IQUB overexpression on breast cancer cells by clone formation assay and Wound healing assay, which indicated that IQUB could promote proliferation and migration of breast cancer cells through activating Akt/GSK3β/β‐catenin signaling pathway (Figure 6C).

In conclusion, our results indicated that the expression of IQUB was increased in breast cancer and positively correlated with malignancy of breast cancer for the first time. IQUB could activate Akt/GSK3β/β‐catenin signaling pathway, promoting proliferation and migration of breast cancer cells. Moreover, our results also suggested that IQUB may be able to regulate Wnt/β‐catenin signaling pathway via another mechanism, except for the Akt/GSK3β/β‐catenin pathway, which is an interesting idea we are focusing on.

## CONFLICT OF INTEREST

The authors have declared that no competing interest exists.
